# Immunotoxicity Monitoring in a Population Exposed to Polychlorinated Biphenyls

**DOI:** 10.3390/ijerph13030295

**Published:** 2016-03-08

**Authors:** Hajo Haase, Astrid Fahlenkamp, Thomas Schettgen, Andre Esser, Monika Gube, Patrick Ziegler, Thomas Kraus, Lothar Rink

**Affiliations:** 1 Medical Faculty, Institute of Immunology, RWTH Aachen University Hospital, Pauwelsstrasse 30, Aachen D-52074, Germany; Haase@TU-Berlin.de; 2 Department of Food Chemistry and Toxicology, Berlin Institute of Technology, Gustav-Meyer-Allee 25, Berlin D-13355, Germany; 3 Department of Anesthesiology, University Hospital Aachen, Pauwelsstrasse 30, Aachen D-52074, Germany; afahlenkamp@ukaachen.de; 4 Department of Occupational and Social Medicine, RWTH Aachen University, Pauwelsstrasse 30, Aachen D-52074, Germany; tschettgen@ukaachen.de (T.S.); anesser@ukaachen.de (A.E.); mgube@ukaachen.de (M.G.); pziegler@ukaachen.de (P.Z.); tkraus@ukaachen.de (T.K.)

**Keywords:** polychlorinated biphenyls, PCB, immune system

## Abstract

The relationship between polychlorinated biphenyl (PCB) burden and several indicators of immune function was investigated as part of the HELPcB (Health Effects in High-Level Exposure to PCB) program, offering bio-monitoring to workers, relatives, and neighbors exposed to PCBs by a German transformers and capacitors recycling company. The present retrospective observational study evaluates the correlation of plasma levels of total PCBs, five indicator congeners (28, 101, 138, 153, 180), and seven dioxin-like congeners (105, 114, 118, 156, 157, 167, 189) with several parameters of immune function. The cross-sectional study was performed immediately after the end of exposure (258 subjects), and one (218 subjects), and two (177 subjects) years later. At the first time point, measurements showed significant positive correlation between congeners with low to medium chlorination and the relative proportion of CD19 positive B-cells among lymphocytes, as well as a negative correlation of PCB114 with serum IgM, and of PCB 28 with suppressor T-cell and NK-cell numbers. Congeners with a high degree of chlorination, in particular PCB157 and 189, were positively associated with expression of the activation marker CD25 on T-cells in the cohort of the second time point. No associations between PCB levels and IFN-y production by T-cells and killing by NK-cells were found. In conclusion, there were several effects on the cellular composition of adaptive immunity, affecting both T- and B-cells. However, the values were not generally outside the reference ranges for healthy adult individuals and did not indicate overt functional immunodeficiency, even in subjects with the uppermost PCB burden.

## 1. Introduction

After observation of elevated polychlorinated biphenyl (PCB) levels in vegetables grown in the vicinity of an industrial area in Dortmund, Germany, it was finally discovered in May of 2010 that inadequate occupational hygiene in a recycling plant for capacitors and transformers had led to a significant body burden of PCBs in workers and their family members, as well as to the exposure of people working or living in the immediate vicinity of the recycling company [1]. As a consequence, the medical surveillance program HELPcB (Health Effects in High-Level Exposure to PCB) was initiated [2]. It provides bio-monitoring to identify potential health risks related to PCB exposure for the affected group of persons. Unusually high exposure levels of the sum of six indicator congeners (PCBs 28, 52, 101, 138, 153, 180) of up to 236 µg/L plasma were observed in some of the workers [1]. This significantly exceeds the background burden previously reported for German subjects with no known exposure to sources of PCB [3]. Exposure also extended to relatives that have not been in direct contact with the contaminated materials [1].

PCBs belong to the persistent organic pollutants, a group of chemicals causing concern due to their widespread accumulation in the environment and food chain, especially in light of their toxic effects. The toxicological profile for PCBs by the International Agency for Research on Cancer (IARC) lists several severe health effects, including the classification of several congeners as carcinogenic to humans (group 1) [4]. Moreover there are data indicating that PCBs are toxic to the nervous, endocrine, and immune systems [5,6].

So far, immunotoxic effects of PCBs in humans were predominantly investigated with a focus on pre- and early postnatal exposure. An elevated risk for respiratory infections due to PCB exposure was identified in Swedish children [7]. Reduced antibody formation in response to vaccination against diphtheria and tetanus was found in a cohort of children from the Faroe Islands. These children had an elevated risk for PCB exposure based on uptake from the consumption of pilot whale blubber [8]. A study in Dutch infants did not confirm a relationship between PCB exposure and antibody production or the incidence of respiratory infections [9]. Instead, it found a positive relationship between current PCB body burden (but not prenatal exposure deduced from maternal plasma levels) and the prevalence of recurrent middle ear infections and Chicken pox, whereas allergies were reduced [10]. On the cellular level, a particular impact on T-cells was found, with PCB exposure resulting in elevated numbers of total and cytotoxic T-cells, and their expression of markers indicating elevated activation (HLA-DR) and formation of memory cells (CD45R0) [10].

In an adult population from the 1970s with occupational PCB exposure, some marginal changes in leukocyte populations were reported, such as slightly increased monocyte numbers, but all values remained within their physiological clinical ranges [11]. Svensson *et al.* investigated the impact of fatty fish consumption, a significant source for persistent organochlorine compounds, including PCB. They observed no differences in lymphocyte levels or subpopulations compared to a control group with low fish consumption, except for a weak reduction of NK-cells, identified by the marker CD56 [12]. These NK-cell numbers were negatively correlated with blood levels of PCB congeners 118 and 126.

So far, HELPcB has reported plasma levels of total PCB and several indicator congeners [1,3] and their relationship to mental illness [13] and quality adjusted life years [14]. The present study investigates the correlation with leukocyte subpopulations and several parameters of immune function for the first three years of cross-sectional examinations.

## 2. Materials and Methods

### 2.1. Study Population

A detailed description of the study population has been published earlier [1]. Subjects involved former employees of a capacitor and transformer recycling company and their family members, as well as employees of surrounding companies, and area residents. The surveillance program was approved by the local ethics committee of the Medical Faculty of the RWTH Aachen University (EK 176/11). A total of 294 adults met the entry requirements of increased PCB blood levels to the HELPcB program. In the present study, children were not included, due to the considerable variation of immunological parameters in early life. Analyses were performed in three cross-sections over three consecutive years (designated t1, t2, and t3). While the majority of subjects (148) took part in all three cross-sections, a substantial number has only participated in one or two cross-sections, due to dropout and/or inclusion at a later time point. Population characteristics are shown in Table 1. Further information has been previously reported [1,2,15].

### 2.2. Quantification of PCB Burden

Plasma was obtained by centrifugation of blood samples for 10 min at 850× g. Subsequently, 3.5 mL of supernatant was transferred to precleaned glass vials and stored at −20 °C until analysis [1]. The PCB burden in blood plasma samples of the participants was measured as previously described [3]. Briefly, plasma samples were deproteinized with formic acid, followed by extraction of PCBs with n-hexane. After a clean-up step on silica-gel columns, samples were analyzed by gas chromatography/electron ionization-mass spectroscopy (GC/EI-MS). Six calibration standards between 0.04 and 3 µg/L were applied. The limit of detection (LOD) based on a signal-to-noise ratio of 3 was determined to be 0.01 μg/L serum for all analytes investigated. The between-day imprecision (n = 58) was determined to be in the range of 2.6%–7.5% for all analytes and the accuracy of our results for the indicator congeners was guaranteed by biannual successful participation in a round robin organized in Germany (www.g-equas.de) [1]. Due to the lipophilic nature of PCBs, PCB data are presented as µg per total serum lipids (TSL). To this end, total cholesterol and triglycerides were measured at the center of laboratory diagnostics of UK Aachen and used to calculate TSL based on the CDC short formula (TSL (mg/dL) = ((2.27 × total cholesterol) + triglycerides + 62.3) × 1.028), assuming a density of serum of 1.028 [16].

### 2.3. Lymphocyte Phenotyping

K+-EDTA anti-coagulated whole blood was incubated for 20 min at room temperature with fluorescently labeled antibody pairs (CD45/CD14, CD3/CD19, CD3/CD4, CD3/CD8, CD3/HLA-DR, CD3/CD25, CD57/CD8, CD3/CD16 + CD56, CD8/CD11b, CD19/CD5, and isotype matched controls IgG1 FITC/IgG2a PE, all from Becton Dickinson, Heidelberg, Germany). Erythrocytes were lysed with BD FACS lysing solution according to the manufacturer’s instructions and leukocytes analyzed by flow cytometry on a FACSCalibur using BD Simulset software for data acquisition and analysis. (Becton Dickinson, Heidelberg, Germany). Total leukocyte content was determined by automatic cell counting using a CASY TT (Schärfe System, Reutlingen, Germany).

### 2.4. Determination of Immune Parameters in Serum

Interleukin 6 as a measure of basal inflammation was determined in t2 by BD OptEIA ELISA (BD Biosciences, Heidelberg, Germany) according to the manufacturer’s instructions. Data were not included in further analyses because the vast majority (>90%) of the serum values were below the detection limit of 4.7 pg/mL.

Immunoglobulin (Ig) levels of IgM, IgG, and IgA were measured at the center of laboratory diagnostics of UK Aachen by a turbidimetric immunoassay according to the manufacturer’s protocols (Roche diagnostics, Mannheim, Germany).

### 2.5. Isolation of Peripheral Blood Mononuclear Cells

Peripheral blood mononuclear cells (PBMC) were isolated from heparinized peripheral venous blood by density gradient centrifugation with Ficoll as previously described [17]. Cells were cultured in RPMI 1640 medium supplemented with 10% low endotoxin fetal calf serum (PAA, Cölbe, Germany) that had been heat-inactivated at 56 °C for 30 min prior to use. Additional medium supplements were 2 mM L-glutamine, 100 U/mL penicillin, and 100 µg/mL streptomycin (all from Lonza, Verviers, Belgium). Cells were kept at 37 °C and saturated humidity in a mixture of 95% air and 5% CO_2_.

### 2.6. Quantification of IFN-γ Production

Heparinized peripheral venous blood was diluted 1:10 with RPMI 1640 and incubated without stimulation or in the presence of either Phytohaemagglutinin (PHA, 10 µg/mL) or Streptococcal pyrogenic exotoxin A (SPEA, 250 ng/mL) at 37 °C and 5% CO_2_ for 3 and 5 days in sterile polypropylene tubes. Supernatants were obtained by centrifugation (10 min, 300× g) and stored at −80 °C. IFN-γ was quantified by ELISA using BD OptEIA Set Human IFN-γ (BD Biosciences, Heidelberg, Germany) according to the manufacturer’s instructions.

### 2.7. NK-Cell Assay

K562 target cells (culture conditions as described above for PBMC) were labeled with carboxyfluorescein diacetate succinimidyl ester (CFDA-SE) (0.1 µM, 37 °C, 10 min) and incubated together with PBMC at a ratio of 1 (K562) to 12.5 (PBMC) at 37 °C and 5% CO_2_ overnight. Dead cells were identified by labeling with propidium iodide (50 µg/mL for 10 min in the dark) on a FACScan flow cytometer (Becton Dickinson, Heidelberg, Germany). NK-cell specific lysis was determined by subtracting the number of dead K562 cells incubated alone from the values obtained in the presence of PBMC.

### 2.8. Measurement of Phagocytosis and Oxidative Burst

Heparinized whole blood was kept at 37 °C or 4 °C (negative control) for 30 min, followed by addition of either *E. coli* (strain BL-21) transformed to express the DsRed gene, or PMA (1 µg/mL), followed by further incubation for 20 min and addition of the redox-sensitive pro-fluorophore dihydrorhodamine 123 (1 µg/mL) to all samples. After incubation for additional 10 min, erythrocytes were lysed with BD FACS lysing solution according to the manufacturer’s instructions and phagocytosis and oxidative burst analyzed by flow cytometry with a FACScan.

### 2.9. Statistical Analysis

Statistical analyses were performed with SPSS for Windows (Version 23, Statistical Package for the Social Sciences, Inc., Chicago, IL, USA) and Graphpad Prism (Version 5.0, GraphPad Software, San Diego, CA, USA). The study group showed a distribution of PCB levels ranging over more than two orders of magnitude. Hence, regression analysis was used to identify potential associations between PCB burden and immunological parameters. First, data were visually inspected for outliers, which were then checked against the raw data to eliminate transcription errors. Normality of all metric variables was checked visually (using histograms) and by the D’Agostino-Pearson omnibus K2 normality test. Not normally distributed datasets (all PCB data and most of the immunological parameters) were normalized by log10-transformation. Data from each cross-section were analyzed by linear correlation analysis calculating the Pearson product-moment correlation coefficient. In a next step, potential confounding effects (age, BMI, sex, smoking habit and pack years, alcohol consumption) were investigated by multiple linear regression analysis, the results of which are shown in separate tables. In case no such table is shown, no significant regression was found by ANOVA (*p* > 0.05).

Correlation analysis was performed for the sum of six indicator congeners (28, 52, 101, 138, 153, and 180), which was taken as a measure for total PCB burden, and the single congeners listed in Table 2. Out of 18 congeners for which plasma levels had been determined, six were below the detection limit of 0.01 μg/L in more than 50% of the subjects and excluded from further analysis. For the remaining datasets, values below the limit of detection were set as LOD/2 (= 0.005 μg/L). PCB plasma levels are presented as medians, and the statistical significance between different groups of subjects was investigated by a two-tailed Mann-Whitney U test or, in case of multiple comparisons, by Kruskal-Wallis test, applied to the non-transformed data.

## 3. Results

**Figure 1:**
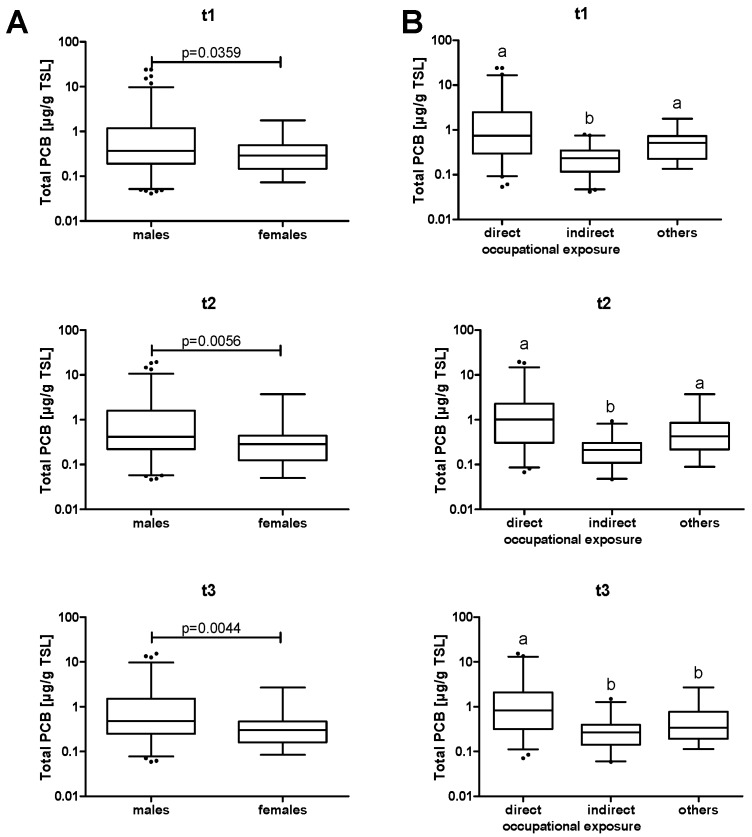
Total PCB burden. Data are shown as box plots depicting the median plasma concentrations. Whiskers represent the 2.5 to 97.5 percentiles. (**A**) Comparison between male and female subjects. Statistical significance of the difference between male and female subjects was calculated by Mann-Whitney U test. (**B**) Comparison between subjects depending on the route of exposure. Statistical significance was calculated by Kruskal-Wallis test. Statistically significant differences are indicated by the absence of shared letters.

The median levels of total plasma PCBs are shown in Table 1 for all three cross-sections. A separate analysis for male and female participants showed a statistically significant higher exposure of males than females (Figure 1A). However, gender was not a major confounding factor in the subsequent analyses. PCB levels were also analyzed according to exposure (Figure 1B). Most subjects were occupationally exposed, with workers from the contaminated recycling plant showing significantly higher plasma levels compared to workers of the immediate vicinity.

**Figure 2:**
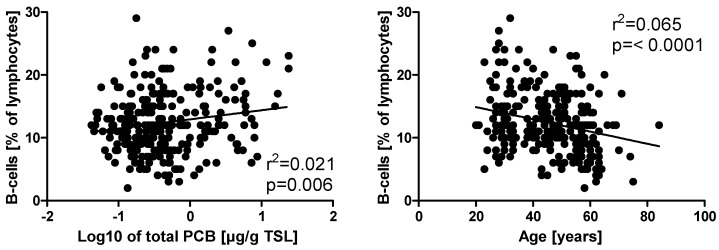
Correlation of the percentage of CD19+ B-cells among lymphocytes with total PCB burden and age. Lines represent the results of linear regression analysis.

Total leukocyte numbers, as well as several surface markers for the major immune cell subpopulations, were measured by flow cytometry (Supplementary Table S1). Subsequently, a correlation analysis was performed between total PCB plasma levels and the results of leukocyte phenotyping for each of the cross-sections (Supplementary Table S2). There was a positive correlation in t1 with the absolute number and relative fraction of cells bearing the surface marker CD19, which designates B-cells, and with the ratio of CD11b negative to positive CD3+CD8+ cells, which indicates the proportion of cytotoxic to suppressor T-cells. Moreover, t2 and t3 showed a correlation between total PCB burden and CD25+ (an activation marker) on T-cells that had not been observed in t1. Finally, in t3, the number of CD3+CD4+ double positive cells (T-helper cells) was positively associated with the PCB content of the plasma. After correction for the potential confounding effects of age, BMI, smoking habits, sex, and alcohol consumption by multiple linear regression analysis, age and smoking were identified as major confounding variables, and only the correlation between plasma PCB levels and the relative number of CD19+ B-cells remained statistically significant (Table 3, Supplementary Table S3). Previous studies have already investigated the influence of several confounding factors on PCB plasma levels in the population of this study, such as age, nationality/migration background, relationship status, and education [14]. Hereby, age was identified as the major confounding variable. In our subjects, we observed a correlation of some immunological parameters with age (Supplementary Table S4). Notably, B-cells showed a negative correlation with age, whereas their levels were positively associated with PCBs (Figure 2).

PCB plasma levels are the sum of several different congeners. Out of a total of 209 potential substances, the levels for 18 congeners were measured separately [1,3]. Highly significant correlations were observed between the different PCBs (data not shown). This was to be expected, because subjects were not exposed to single congeners, but to mixtures, leading to combined exposure. Toxicodynamic properties are affected by the degree of chlorination, and the half-life of congeners increases with the number of chlorine atoms in the molecule [18]. Accordingly, PCB congeners with a lower degree of chlorination were even negatively correlated with age, whereas six out of seven highly chlorinated congeners showed a positive association (Supplementary Figure S1, Table S5).

An analysis of potential correlations was performed between plasma levels of the different congeners and lymphocyte surface markers for each cross-section (Supplementary Tables S6–S10). The observed effects were dependent on the degree of PCB chlorination. The positive relationship of PCB levels with B-cell numbers (CD19+) in t1, which was not significant for total PCBs after adjustment for confounding effects, was significant for congeners 101 and 105 (Table 3, Supplementary Tables S6 and S7). Moreover, the association between relative B-cell numbers among lymphocytes was confirmed for congeners with a low to intermediate (three to six) number of chlorine atoms, whereas highly chlorinated (seven Cl) PCBs were not associated.

As a functional parameter for B-cells, serum antibody levels were determined (Supplementary Table S12). IgM negatively correlated with all PCB congeners in t1. This effect was reduced in t2 and absent in t3. After multiple linear regression, PCB 114 in t1 was the only remaining significant correlation (Table 3, Supplementary Table S13). For the other immunoglobulins, we found no correlation of PCBs with IgG or IgA. Notably, no significant correlations between antibody levels and any of the B-cell parameters were found (data not shown).

Several correlations of PCB congeners were observed with T-cells (Tables S6–S10). T-helper-cells (CD3+CD4+) were positively associated with PCB levels in t1 and t3, although with changing congeners and not statistically significant according to multiple linear regression (Supplementary Tables S7 and S11). Notably, the association between CD3+CD8+CD11b+ suppressor T-cells and PCB28 was highly significant in t1. In addition, a positive correlation of total PCBs had already been observed for CD25+ T-cells in t2 and t3 (Supplementary Table S2). This effect is predominantly associated with highly chlorinated congeners and significant for the congeners 157 and 189 in t2 (Table 3, Supplementary Tables S8 and S9).

As a measure of T-cell function, the production of IFN-γ in response to treatment with the T-cell-mitogen PHA and the superantigen SPEA was measured (Supplementary Table S14). No association between IFN-γ and any PCB levels were identified in cross-section t1 and production of IFN-γ was not further investigated in the following years. For t3 the numbers of T-cells expressing CD45RA (a marker for naïve cells) and CD45R0 (a marker for memory cells) were added to the panel of T-cell markers. The percentage of memory cells were positively associated with total PCBs and congeners 153 to 189 (Supplementary Table S15). However, those associations mostly resulted from covariation with age and were not significant according to multiple linear regression.

In the present study, there was a statistically significant negative association of CD16+CD56+ cells with PCB28 in t1 (Table 3, Supplementary Tables S6 and S7). Hence, in t3 a more detailed investigation of NK-cell function was included. Yet, there was no longer any correlation between PCB plasma levels and CD16+CD56+ NK-cells in subsequent cross-sections (Supplementary Tables S7 and S8), and some weak associations of various congeners with CD3+CD16+/CD56+ NKT-cells (Supplementary Table S15) were observed, but not significant according to multiple linear regression. In addition to measurement of total NK- and NKT-cell numbers, a killing assay with K562 cells was performed in order to control for a potential functional impact on NK-cell activity. No significant correlations were detected, suggesting that PCB levels in our subjects had no functional impact on NK-cells (Supplementary Table S15).

## 4. Discussion

Total PCB blood levels in the present study covered a range of nearly three orders of magnitude, ranging from values comparable to those found in the general German population [3] for the least contaminated participants to maximum exposure levels of up to 24 µg/g TSL, with means around 0.4 µg/g TSL. This is very similar to the data observed in a study with Italian subjects with occupational exposure to PCB [19]. In contrast, the means are lower than those reported by Seegal *et al.* in former capacitator workers, showing geometric means around 1 µg/g of lipids [18]. This was most likely due to the fact that the latter study focused on subjects with direct occupational exposure, whereas our study also included relatives and workers from companies surrounding the source of contamination.

Despite the observed association between several immune parameters and PCB burden, even the highly exposed individuals did not show indications for any overt immunodeficiency. Most values were within the respective reference ranges and a statistical analysis of the number of infections reported by the subjects and PCB plasma levels in t1 did also not yield significant correlations (data not shown). Hence, our results gave no indication for overt immunotoxicity manifesting in drastically elevated probability for infectious diseases. A similar conclusion was also drawn by Lawton *et al.*, who had investigated the effects of PCBs in capacitor workers [11]. They observed statistically significant changes in some leukocyte subpopulations, in particular the absolute number and relative fraction of monocytes, but parameters remained within their normal clinical ranges with no acute pathological effects. Admittedly, measurement of a high number of various different immune parameters increases the risk for observing effects based on type 1 error. Still, there are a high number of potential targets for PCBs in the immune system, which have to be considered for an analysis to be sufficiently comprehensive. E.g., further investigations have shown accelerated telomere shortening in lymphocytes of PCB exposed workers (manuscript in revision). Short telomeres could be an indicator of premature immunosenescence. This demonstrates that, in addition to the parameters investigated in this study, which focused on previously reported immunological effects, there are yet other potential aspects of immunotoxicity of PCBs to be considered.

In the present study, one finding was the association of low to medium chlorinated PCB congener plasma levels with relative B-cell numbers in cross-section t1. Several previous human studies had found B-cell numbers to be unaffected by PCBs [7,12,20], whereas one group mentioned a negative correlation in postnatally exposed infants three months of age, but no correlation to prenatal exposure [9,10]. Still, PCB burden was negatively associated with reduced production of IgM and IgG antibodies against sheep red blood cells in monkeys [21], and with reduced antibody formation in response to vaccination in children [8], suggesting a potential functional impairment of B-cells or their interaction with T-helper-cells. In our study, this was only confirmed for IgM, which showed a negative correlation with PCB114 in t1. Because the formation of IgM does not require T-helper-cells, this indicates a direct effect of PCB exposure on B-cell functionality.

Impacts of PCBs on thymus size, T-cell subsets, and T-cell activity were observed in several animal models, mostly rodents and monkeys [5], and also in PCB-exposed workers [19]. In the present study, effects comprised positive correlations between PCBs with the number of suppressor T-cells (PCB28 in t1) and the expression of the T-cell activation marker CD25 (PCBs157 and 189 in t2). This indicates another potential impact of PCBs on the adaptive immune system. In this context, it should also be noted that the median levels of CD3+CD25+ cells for the general study population were above the reference ranges in Table S1. An association between PCBs and CD25 expression on T-cells had been previously investigated [12]. The percentage was higher in consumers of fatty fish, who showed elevated levels of PCB burden, but data were not statistically significant. Still, this might be due to that fact that only relatively small numbers of 23 fish eaters and 20 controls had been investigated [12]. Slightly higher levels of CD45R0+ T-cells have been reported in the same study, but these effects were also not statistically significant [12], comparable to the results in the present study.

The presence of immune cells does not equal the absence of functional impairment, as shown above for IgM production and B-cells. Several other aspects of functionality were tested, including IFN-γ production by T-cells and killing by NK-cells. Hereby, the present study only monitored some of the most important immune parameters. Yet, the complexity of the immune system does not allow for a complete monitoring of all functions relevant for immunity. Several animal studies have shown enhanced responses to the T-cell mitogen PHA [5], but not even a trend was observed when the correlation between PCBs and IFN-γ production in response to PHA or SPEA was investigated. In the literature, NK-cells showed a significant negative correlation between NK-cell numbers and PCB congeners 118 and 126 [12]. In contrast, no statistically significant effects, or even an elevation of NK-cell activity, were found in rhesus monkeys [21,22]. In the present study, NK-cell numbers and killing activity were not significantly correlated with PCB burden. Another function that has been reported to be affected by PCBs in human leukocytes is phagocytosis [23]. Hence, additional experiments by flow cytometry were performed, investigating the cells’ capacity for phagocytosis of bacteria and killing by the oxidative burst. The cells were functional, generally above 95% were capable of performing phagocytosis and oxidative burst, and no indication for an impact of PCB plasma levels was observed (data not shown).

It seems counterintuitive that median plasma levels increased from t1 to t3 (Figure 1), despite the fact that exposure was stopped as soon as the contamination had been detected. However, results of the bio-monitoring program were communicated to the subjects after each round of analyses. This led to a selective dropout of participants with lower PCB levels, who did no longer feel the need for further bio-monitoring. Consequentially, no noteworthy changes of total PCB plasma content were observed when only the data of a subgroup of participants were analyzed, which had participated in all three cross-sections. Still, these values did not decrease either, which is probably based on the fact that the highest median congener concentrations were those of PCBs 138, 153, 180, which have a long half-life due to their high degree of chlorination. Due to this long half-life period and their persistence in food chain and environment, these congeners accumulate in humans [24]. The dropout of participants also had an impact on data analysis. While an investigation in three consecutive years might seem to be ideally suited for a longitudinal study, data were analyzed as three separate cross-sections due to the fact that less than 60% of the subjects participated in all three of these cross-sections. Notably, non-random dropout, predominantly of subjects with low level exposure, bears the danger of selection bias by leaving a disproportionate fraction of highly exposed subjects in the later cross-sections. In the present study, the number and statistical significance of correlations between PCB levels and immune parameters declined from t1 to t3, indicating that the combined effects from an end of PCB exposure and a loss of statistical power due to the reduction of the study group size exceeded potential effects of an accumulation of highly exposed subjects, which might promote the detection of stronger associations.

There were far more males than females in the study group, and the latter showed a significantly lower PCB burden. It has previously been reported that women and men have differences in their blood congener profiles despite similar dietary intake [25], indicating a potential difference in metabolism, and that *in utero* exposure to PCBs may have gender-specific toxic effects in mice [26]. This may result from a differential metabolism, because rat liver tissue slices showed differences in the formation of hydroxylated PCB metabolites by cytochrome P450 monooxigenases [27]. However, in the present study the vast majority of the employees were male, and exposure of female individuals occurred mostly indirectly, for example through their spouses or fathers. Hence, the differences are more likely to originate from differences in exposure rather than metabolism. Notably, the proportion of women increased from 13.2% in t1 to 17.5% in t3. This resulted mainly from disproportionate inclusion of women into the later cross-sections rather than from lower dropout rates, because the fraction of female participants that completed all three cross-sections was 14.2%.

Children are particularly affected by exposure to PCBs, showing elevated incidence of infections [7,8,10,20]. In the present study, only a comparatively small number of children were among the subjects. These were not included in data analysis because their reference values differ considerably from those of adults and are not comparable. Moreover, the total number of 14 children was too small for a subgroup analysis with sufficient statistical power.

Even the strongest associations had correlation coefficients that rarely exceeded 0.35, which is generally considered a weak correlation. Yet, there is a high degree of variability in immune parameters between different individuals, and a more stringent correlation is not to be expected due to many other influencing factors. It was noticeable that the overall number and strength of correlations declined from t1 to t3, possibly resulting from an end of the contact with PCBs. Exposure was stopped as soon as the contamination was detected. This should at least have led to reduced levels of congeners with a lower degree of chlorination, whereas highly chlorinated PCBs are more persistent [18]. This corresponds to the observation that the effect on B-cells (predominantly associated with congeners with low to intermediate chlorination), and on suppressor T-cells and NK-cells (associated with PCB28, which has three chlorine atoms), were only observed in t1, whereas CD25 expression, which was predominantly associated with PBC157 and PCB189, became significant in t2. Yet, at least two other factors might influence these results. First, the selective dropout of participants with low PCB burden led to an increase of median total PCB levels. Second, the declining number of study subjects reduced the statistical power of the analyses. Nevertheless, there are relevant confounding variables when investigating the impact of PCB burden on the immune system, most importantly age, smoking, and alcohol consumption [19] and their effects were clearly seen in the present study, indicating that data analysis had the potential for identifying existing relationships in all three cross-sections.

Correlations between plasma PCB levels and immunological observations are no proof for a causal relationship. It is known that different congeners have diverse toxicological properties [28]. Due to the strong correlation between plasma levels among the different congeners it is difficult to identify a single one that is responsible for immunomodulation, if it even exists. Some polychlorinated dibenzo-p-dioxins are highly toxic, with most toxic effects being mediated through the aromatic hydrocarbon receptor [29]. They are carcinogenic and show toxicity toward several organs, including the immune system. Similar toxicological properties were also reported for 12 out of the total of 209 PCB congeners, which have therefore been designated “dioxin-like PCBs” [29,30]. None of our analyses suggested differences between non-dioxin-like indicator congeners (PCBs 28, 101, 138, 153, and 180) and dioxin-like congeners (PCBs 105, 114, 118, 156, 157, 167, and 189).

PCB metabolism is rather slow, and should lead to improved excretion due to higher hydrophilic properties. This suggests that a large part of the toxic effects of PCBs is mediated directly by these compounds. Nevertheless, it is also possible that effects could be caused by metabolites, such as hydroxyl or methyl sulfone PCBs, some of which show considerable toxicity [31]. Oxidative phase 1 metabolism could also lead to reactive epoxides, which might form adducts with biological macromolecules. Oxidation, mainly by cytochrome P450 monooxigenases, leads primarily to hydroxyl-PCBs. From the 209 congeners, 837 mono-hydroxylated metabolites can be formed, which then could undergo phase 2 reactions, such as glutathionylation or glucoronidation, or further oxidation and introduction of one or more additional hydroxyl moieties into the molecule. This leads to a virtually unlimited number of metabolites, each with different metabolic fate and toxicity [32]. In general, the reactivity depends on the degree and pattern of chlorination, whereby less chlorinated PCB show higher reactivity and thereby faster metabolization, and highly chlorinated ones show less reactivity and a tendency to accumulate in adipose tissue. The complex chemistry and toxicology has recently been summarized in great detail by Grimm *et al.* [32]. In addition, it should also be noted that the observed effects could have resulted from co-exposure to substances that were also contained in the contaminated materials, but not analyzed in the plasma samples. Most importantly, other organochlorine compounds, such as dioxins and furans, have well documented immunotoxic effects [30]. Co-exposure to some of these substances could have also affected our results. Pilot measurements demonstrated the existence of elevated concentrations of polychlorinated dibenzo-p-dioxins and dibenzofurans in the plasma of several members of a subgroup of 23 exposed workers from the HELPcB program [15].

## 5. Conclusions

Some immune parameters were significantly correlated with certain PCB congener levels. Here, lymphocytes were particularly affected. In t1, higher relative numbers of B-cells were associated with low to intermediately chlorinated PCBs, and IgM levels correlated with PCB114. Moreover, some T-cell populations showed correlations, most strongly T-suppressor cells with PCB28 in t1. In contrast, no significant effects on NK-cell numbers and functions were seen, even though these had also been reported in the literature to be modulated by PCB exposure. Based on the results obtained in the first three cross-sections, no acute immunotoxicity has been found. Yet, the major future task for the HELPcB program with respect to immunity will be to monitor long term health impacts, because some effects may occur on a significantly greater timescale than the ones investigated so far.

## Figures and Tables

**Table 1:** Study population.

Parameters	t1	t2	t3
Number of subjects	258	218	177
Sample collection interval	08/2010–06/2011	09/2011–06/2012	08/2012–02/2013
Male/female	224/34	182/36	146/31
Median total PCB (min/max) (µg/g TSL)	0.363 (0.041/24.07)	0.367 (0.047/19.59)	0.421 (0.059/15.47)
Age, mean (min/max) (years)	45.4 (20/84)	46.2 (21/85)	47.9 (25/86)
Mean body mass index	27.7	27.9	28.0
Exposure (direct/indirect/others) ^a^	133/109/16	120/76/22	102/57/18
Smoking habit (never/current/former)	56/129/73	59/96/63	46/76/55
Subjects reporting daily alcohol consumption	66	50	42

^a^ refers to occupational exposure of subjects working in the contaminated recycling plant (direct) and in surrounding companies within the same industrial area (indirect). The remainder (others) was exposed by various non-occupational ways.

**Table 2:** PCB congeners.

Congener	Name	No. of Cl	Included
28	2, 4, 4’-trichlorobiphenyl	3	Yes
52	2, 2’, 5, 5’-tetrachlorobiphenyl	4	No
77	3, 3', 4, 4’-tetrachlorobiphenyl	4	No
81	3, 4, 4', 5-tetrachlorobiphenyl	4	No
101	2, 2’, 4, 5, 5'-pentachlorobiphenyl	5	Yes
105	2, 3, 3’, 4, 4’-pentachlorobiphenyl	5	Yes
114	2, 3, 4, 4’, 5-pentachlorobiphenyl	5	Yes
118	2, 3’, 4, 4’, 5-pentachlorobiphenyl	5	Yes
123	2’, 3, 4, 4’, 5-pentachlorobiphenyl	5	No
126	3, 3’, 4, 4’, 5-pentachlorobiphenyl	5	No
138	2, 2’, 3, 4, 4’, 5’-hexachlorobiphenyl	6	Yes
153	2, 2’, 4, 4’, 5, 5’-hexachlorobiphenyl	6	Yes
156	2, 3, 3’, 4, 4’, 5-hexachlorobiphenyl	6	Yes
157	2, 3, 3’, 4, 4’, 5’-hexachlorobiphenyl	6	Yes
167	2, 3, 4, 4’, 5, 5’-hexachlorobiphenyl	6	Yes
169	3, 3’, 4, 4’, 5, 5’-hexachlorobiphenyl	6	No
180	2, 2’, 3, 4, 4’, 5, 5’-heptachlorobiphenyl	7	Yes
189	2, 3, 3’, 4, 4’, 5, 5’-heptachlorobiphenyl	7	Yes

**Table 3:** Overview of significant correlations between PCB levels and immunological parameters.

Dependent Variable	Cross-Section	Congener	R^2^	β	*p*
CD19 (%)	1	Total PCB	0.165	0.169	0.017
CD19 (µL^−1^)	1	101	0.170	0.166	0.026
	1	105	0.165	0.143	0.049
CD19 (%)	1	28	0.165	0.172	0.016
	1	101	0.173	0.202	0.007
	1	105	0.178	0.210	0.004
	1	118	0.174	0.196	0.006
	1	138	0.162	0.161	0.023
	1	153	0.159	0.150	0.034
	1	156	0.163	0.164	0.023
	1	157	0.158	0.148	0.038
	1	167	0.165	0.168	0.017
CD3+CD8+CD11b+ (µL^−1^)	1	28	0.039	−0.250	0.001
CD16+CD56+ (µL^−1^)	1	28	0.054	−0.151	0.046
IgM	1	114	0.039	−0.205	0.008
CD3+CD25+ (µL^−1^)	2	157	0.297	0.157	0.037
	2	189	0.295	0.155	0.049

Adjusted proportion of the variance explained by the model (Adj. R^2^), standardized estimate (β), and level of significance (*p*) from multiple linear regressions with the potential confounders age, BMI, smoking habits, sex, and alcohol consumption. Only data for immunological parameters with statistically significant correlations with PCB levels (*p* < 0.05) are shown. For complete results see supplementary materials, Supplementary Tables S2, S6–S12.

## Supplementary Materials

The following are available online at www.mdpi.com/1660-4601/13/3/295/s1, Figure S1: Correlation of total PCB and selected congeners with age in t1, Table S1: Summary of immunophentyping data; Table S2: Correlation analysis of total PCB with immunophentyping data, Table S3: Multiple linear regression for significant correlations from Table S2, Table S4: Correlation analysis of age with lymphocyte markers, Table S5: Correlation between age and PCB burden, Table S6: Pearson correlation analysis of lymphocyte markers with PCB congener plasma levels in t1, Table S7: Multiple linear regression for significant correlations from table S6, Table S8: Pearson correlation analysis of lymphocyte markers with PCB congener plasma levels in t2, Table S9: Multiple linear regression for significant correlations from Table S8, Table S10: Pearson correlation analysis of lymphocyte markers with PCB congener plasma levels in t3, Table S11: Multiple linear regression for significant correlations from table S10, Table S12: Correlation analysis of PCB congeners with antibody levels; Table S13: Multiple linear regression for significant correlations from table S12; Table S14: Correlation analysis of PCB congeners with IFN-γ production in t1, Table S15: Correlation analysis of PCB congeners with markers of T- and NK-cell activation in t3.

## Abbreviations

The following abbreviations are used in this manuscript:

CFDA-SE: carboxyfluorescein diacetate succinimidyl ester
GC/EI-MS: gas chromatography/electron ionization–mass spectroscopy
HELPcB: Health Effects in High-Level Exposure to PCB
IARC: International Agency for Research on Cancer
PBMC: Peripheral blood mononuclear cells
PCB: polychlorinated biphenyls
PHA: Phytohaemagglutinin
SPEA: Streptococcal pyrogenic exotoxin A
TSL: total serum lipids

## References

[1] Schettgen T., Gube M., Esser A., Alt A., Kraus T. (2012). Plasma polychlorinated biphenyls (PCB) levels of workers in a transformer recycling company, their family members, and employees of surrounding companies. J. Toxicol. Environ. Heal. Part A.

[2] Kraus T., Gube M., Lang J., Esser A., Sturm W., Fimm B., Willmes K., Neulen J., Baron J. M., Merk H. (2012). Surveillance program for former PCB-exposed workers of a transformer and capacitor recycling company, family members, employees of surrounding companies, and area residents—Executive summary. J. Toxicol. Environ. Heal. Part A.

[3] Schettgen T., Gube M., Alt A., Fromme H., Kraus T. (2011). Pilot study on the exposure of the German general population to non-dioxin-like and dioxin-like PCBs. Int. J. Hyg. Environ. Health.

[4] International Agency for Research on Cancer (IARC) Polychlorinated Biphenyls and Polybrominated Biphenyls. http://monographs.iarc.fr/ENG/Monographs/vol107/index.php.

[5] Agency for Toxic Substances and Diseases Registry ATSDR Toxicological Profile for Polychlorinated Biphenyls (PCBs) 2000. http://www.atsdr.cdc.gov/toxprofiles/tp17.pdf.

[6] Agency for Toxic Substances and Diseases Registry ATSDR Toxicological Profile for Polychlorinated Biphenyls (PCBs) (Addendum) 2011. http://www.atsdr.cdc.gov/toxprofiles/pcbs_addendum.pdf.

[7] Glynn A., Thuvander A., Aune M., Johannisson A., Darnerud P.O., Ronquist G., Cnattingius S. (2008). Immune cell counts and risks of respiratory infections among infants exposed pre- and postnatally to organochlorine compounds: A prospective study. Environ. Health.

[8] Heilmann C., Budtz-Jørgensen E., Nielsen F., Heinzow B., Weihe P., Grandjean P. (2010). Serum concentrations of antibodies against vaccine toxoids in children exposed perinatally to immunotoxicants. Environ. Health Perspect..

[9] Weisglas-Kuperus N., Sas T.C., Koopman-Esseboom C., Van der Zwan C.W., De Ridder M.A., Beishuizen A., Hooijkaas H., Sauer P.J. (1995). Immunologic effects of background prenatal and postnatal exposure to dioxins and polychlorinated biphenyls in Dutch infants. Pediatr. Res..

[10] Weisglas-Kuperus N., Patandin S., Berbers G.A., Sas T.C., Mulder P.G., Sauer P.J., Hooijkaas H. (2000). Immunologic effects of background exposure to polychlorinated biphenyls and dioxins in Dutch preschool children. Environ. Health Perspect..

[11] Lawton R.W., Ross M.R., Feingoldt J., Brown J.F. (1985). Effects of PCB exposure on biochemical and hematological findings in capacitor workers. Environ. Health Perspect..

[12] Svensson B.G., Hallberg T., Nilsson A., Schutz A., Hagmar L. (1994). Parameters of immunological competence in subjects with high consumption of fish contaminated with persistent organochlorine compounds. Int. Arch. Occup. Environ. Health.

[13] Gaum P.M., Esser A., Schettgen T., Gube M., Kraus T., Lang J. (2014). Prevalence and incidence rates of mental syndromes after occupational exposure to polychlorinated biphenyls. Int. J. Hyg. Environ. Health.

[14] Esser A., Gaum P.M., Schettgen T., Kraus T., Gube M., Lang J. (2014). Effect of occupational polychlorinated biphenyls exposure on quality-adjusted life years over time at the HELPcB surveillance program. J. Toxicol. Environ. Heal. Part A.

[15] Wittsiepe J., Wilhelm M., Kraus T. (2012). Levels of polychlorinated dibenzo-p-dioxins and dibenzofurans (PCDD/F) in blood samples of occupationally exposed workers from a transformer recycling plant in Dortmund, Germany—Initial findings. J. Toxicol. Environ. Heal. Part A.

[16] Bernert J.T., Turner W.E., Patterson D.G., Needham L.L. (2007). Calculation of serum “total lipid” concentrations for the adjustment of persistent organohalogen toxicant measurements in human samples. Chemosphere.

[17] Haase H., Ober-Blöbaum J.L., Engelhardt G., Hebel S., Heit A., Heine H., Rink L. (2008). Zinc signals are essential for lipopolysaccharide-induced signal transduction in monocytes. J. Immunol..

[18] Seegal R.F., Fitzgerald E.F., Hills E.A., Wolff M.S., Haase R.F., Todd A.C., Parsons P., Molho E.S., Higgins D.S., Factor S.A. (2011). Estimating the half-lives of PCB congeners in former capacitor workers measured over a 28-year interval. J. Expo. Sci. Environ. Epidemiol..

[19] D’Errico M.N., De Tullio G., Di Gioacchino M., Lovreglio P., Basso A., Drago I., Serra R., Apostoli P., Vacca A., Soleo L. (2012). Immune effects of polychlorinated biphenyls, smoking and alcohol. Int. J. Immunopathol. Pharmacol..

[20] Dewailly É., Ayotte P., Bruneau S., Gingras S., Belles-Isles M., Roy R. (2000). Susceptibility to infections and immune status in Inuit infants exposed to organochlorines. Environ. Health Perspect..

[21] Arnold D.L., Bryce F., Mes J., Tryphonas H., Hayward S., Malcolm S. (1999). Toxicological consequences of feeding PCB congeners to infant rhesus (*Macaca mulatta*) and cynomolgus (*Macaca fascicularis*) monkeys. Food Chem. Toxicol..

[22] Tryphonas H., Luster M.I., White K.L., Naylor P.H., Erdos M.R., Burleson G.R., Germolec D., Hodgen M., Hayward S., Arnold D.L. (1991). Effects of PCB (AROCLOR® 1254) on non-specific immune parameters in Rhesus (*Macaca mulatta*) monkeys. Int. J. Immunopharmacol..

[23] Levin M., Morsey B., Mori C., Nambiar P.R., De Guise S. (2005). Non-coplanar PCB-mediated modulation of human leukocyte phagocytosis: A new mechanism for immunotoxicity. J. Toxicol. Environ. Health. A.

[24] Schettgen T., Alt A., Esser A., Kraus T. (2015). Current data on the background burden to the persistent organochlorine pollutants HCB, p, p′-DDE as well as PCB 138, PCB 153 and PCB 180 in plasma of the general population in Germany. Int. J. Hyg. Environ. Health.

[25] Knutsen H.K., Kvalem H.E., Haugen M., Meltzer H.M., Brantsaeter A.L., Alexander J., Päpke O., Liane V.H., Becher G., Thomsen C. (2011). Sex, BMI and age in addition to dietary intakes influence blood concentrations and congener profiles of dioxins and PCBs. Mol. Nutr. Food Res..

[26] Rashid C.S., Carter L.G., Hennig B., Pearson K.J. (2013). Perinatal polychlorinated biphenyl 126 exposure alters offspring body composition. J. Pediatr. Biochem..

[27] Wu X., Kania-Korwel I., Chen H., Stamou M., Dammanahalli K.J., Duffel M., Lein P.J., Lehmler H.-J. (2013). Metabolism of 2, 2’, 3, 3’, 6, 6’-hexachlorobiphenyl (PCB 136) atropisomers in tissue slices from phenobarbital or dexamethasone-induced rats is sex-dependent. Xenobiotica.

[28] Mcfarland V.A., Clarke J.U. (1989). Environmental occurrence, abundance, and potential toxicity of polychlorinated biphenyl congeners: Considerations for a congener-specific analysis. Environ. Health Perspect..

[29] Giesy J.P., Kannan K. (1998). Dioxin-like and non-dioxin-like toxic effects of polychlorinated biphenyls (PCBs): Implications for risk assessment. Crit. Rev. Toxicol..

[30] Larsen J.C. (2006). Risk assessments of polychlorinated dibenzo-p-dioxins, polychlorinated dibenzofurans, and dioxin-like polychlorinated biphenyls in food. Mol. Nutr. Food Res..

[31] Quinete N., Schettgen T., Bertram J., Kraus T. (2014). Occurrence and distribution of PCB metabolites in blood and their potential health effects in humans: A review. Environ. Sci. Pollut. Res..

[32] Grimm F.A., Hu D., Kania-Korwel I., Lehmler H.-J., Ludewig G., Hornbuckle K.C., Duffel M.W., Bergman Å., Robertson L.W. (2015). Metabolism and metabolites of polychlorinated biphenyls. Crit. Rev. Toxicol..

