# Supplementary material for: Immunotoxicity Monitoring in a Population Exposed to Polychlorinated Biphenyls
Source: Int J Environ Res Public Health. 2016 Mar 8;13(3):295. doi: 10.3390/ijerph13030295 (PMC4808958; doi:10.3390/ijerph13030295)
Supplement: Supplementary file 1 [file ijerph-13-00295-s001.pdf]

Supplemental Materials: Immunotoxicity Monitoring in a Population Exposed to Polychlorinated Biphenyls

Hajo Haase, Astrid Fahlenkamp, Thomas Schettgen, Andre Esser, Monika Gube, Patrick Ziegler, Thomas Kraus and Lothar Rink

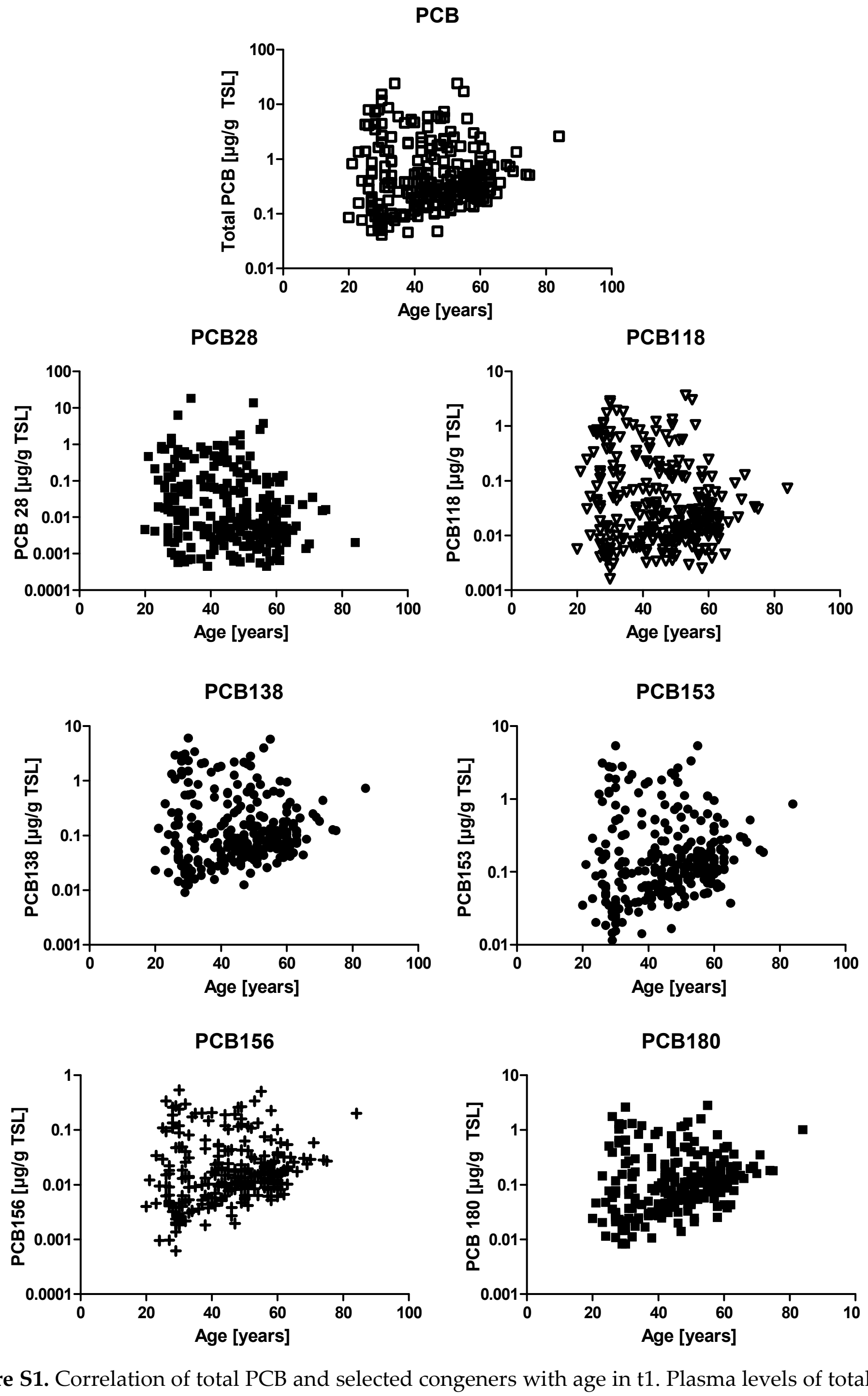

Figure S1. Correlation of total PCB and selected congeners with age in t1. Plasma levels of total PCB and the six congeners with the highest median plasma levels (PCB28, PCB118, PCB138, PCB153, PCB156, PCB180) are depicted in relation to age.

Table S1. Summary of immunophentyping data.

	t1	t2	t3	Reference Range
	**Median (25%; 75%)**	**Median (25%; 75%)**	**Median (25%; 75%)**	**(Min–Max)**
Leukocytes (μL^{-1})	7000 (5700; 8800)	6500 (5300; 8100)	7000 (5900; 8400)	4600–7100
Lymphocytes (μL^{-1})	1981 (1634; 2450)	1800 (1477; 2223)	1966 (1659; 2452)	1600–2400
Lymphocytes (% of leukocytes)	29.0 (23.0; 52.0)	28.0 (23.8; 33.0)	29.5 (24.6; 33.9)	28.0–39.0
CD19+ (μL^{-1})	233 (162; 329)	228 (168; 328)	274 (189; 353)	200–400
CD19+ (% of lymphocytes)	12.0 (9.0; 15.0)	13.0 (10.0; 16.0)	13.0 (11.0; 16.0)	11.0–16.0
CD19+CD5+ (% of CD19+)	48.0 (38.8; 58.0)	54.0 (44.0; 64.0)	47.0 (38.0; 56.0)	≤20.0
CD3+ (μL^{-1})	1371 (1137; 1761)	1259 (990; 1521)	1331 (1075; 1693)	1100–1700
CD3+ (% of lymphocytes)	71.0 (66.0; 75.0)	70.0 (65.0; 75.0)	68.0 (63.0; 73.0)	67.0–76.0
CD3+CD4+ (μL^{-1})	863 (705; 1135)	776 (605; 1014)	846 (674; 1055)	700–1100
CD3+CD8+ (μL^{-1})	507 (363; 643)	436 (315.0; 620.5)	497 (339; 667)	500–900
CD3+CD4+/CD3+CD8+	1.83 (1.32; 2.38)	1.81 (1.32; 2.46)	1.74 (1.25; 2.38)	1.00–1.50
CD3+CD8+CD11b-/CD3+CD8+CD11b+	1.10 (0.70; 1.80)	2.20 (1.40; 3.13)	1.70 (1.10; 2.60)	≤3.00
CD3+CD8+CD11b- (μL^{-1})	331 (227; 459)	385 (291; 531)	397 (273; 551)	n.a.
CD3+CD8+CD11b+ (μL^{-1})	302 (195; 427)	184 (144; 262)	240 (170; 329)	n.a.
CD16+CD56+ (μL^{-1})	313 (212; 436)	283 (210; 378)	332 (247; 424)	200–400
CD3+HLA_DR+ (% of CD3+)	11.9 (8.3; 16.9)	12.7 (9.2; 17.1)	11.6 (8.8; 16.0)	8.0–15.0
CD3+CD25+ (% of CD3+)	39.7 (31.1; 47.4)	33.5 (26.5; 40.9)	36.2 (28.1; 44.35)	13.0–24.0

Data are shown as medians with 25% and 75% percentile. N.a., not available.

Table S2. Correlation analysis of total PCB with immunophentyping data.

	t1		t2		t3	
	r	*p*	*r*	*p*	*r*	*p*
Leukocytes (μL^{-1})	0.083	0.182	0.069	0.313	0.076	0.314
Lymphocytes (μL^{-1})	0.089	0.156	0.090	0.186	0.141	0.062
Lymphocytes (% of leukocytes)	−0.038	0.548	0.027	0.693	−0.087	0.249
CD19+ (μL^{-1})	0.153	0.014	0.084	0.216	0.087	0.252
CD19+ (% of lymphocytes)	0.171	0.006	0.056	0.411	0.015	0.838
CD19+CD5+ (% of CD19+)	−0.026	0.675	−0.126	0.064	−0.071	0.348
CD3+ (μL^{-1})	0.088	0.160	0.086	0.206	0.160	0.033
CD3+ (% of lymphocytes)	−0.022	0.721	0.024	0.723	0.093	0.219
CD3+CD4+ (μL^{-1})	0.092	0.141	0.089	0.193	0.145	0.053
CD3+CD8+ (μL^{-1})	0.026	0.673	0.020	0.766	0.116	0.123
CD3+CD4+/CD3+CD8+	0.046	0.460	0.049	0.472	−0.013	0.864
CD3+CD8+CD11b-/CD3+CD8+CD11b+	0.144	0.021	0.005	0.942	0.040	0.594
CD3+CD8+CD11b- (μL^{-1})	0.109	0.080	0.038	0.576	0.145	0.054
CD3+CD8+CD11b+ (μL^{-1})	−0.116	0.063	0.027	0.697	0.091	0.229
CD16+CD56+ (μL^{-1})	−0.035	0.572	0.033	0.626	−0.034	0.657
CD3+HLA_DR+ (% of CD3+)	−0.011	0.861	0.032	0.640	0.016	0.834
CD3+CD25+(% of CD3+)	0.025	0.691	0.158	0.020	0.152	0.043

Pearson-correlation between total plasma PCB levels and selected immune parameters, expressed by correlation coefficients and *p* values. Statistically significant positive correlations ($p < 0.05$) are highlighted in blue color.

Table S3. Multiple linear regression for significant correlations from Table S2.

Dependent Variable	t	Adj. R^2		Independent Variables						
				PCB	Age	Smoking	Pack Years	Sex	BMI	Alcohol
CD19 (μL⁻¹)	1	0.161	β	0.121	−0.249	0.206	0.139	−0.057	0.153	−0.127
			p	0.087	0.006	0.009	0.095	0.424	0.033	0.076
CD19 (%)	1	0.165	β	0.169	−0.266	0.134	0.095	−0.070	0.160	−0.168
			p	0.017	0.003	0.087	0.252	0.326	0.026	0.018
CD3+CD25+ (% of CD3+)	2	0.292	β	0.134	0.354	0.237	0.285	0.123	−0.074	−0.011
			p	0.070	0.000	0.003	0.001	0.107	0.327	0.876
CD3+CD25+ (% of CD3+)	3	0.316	β	0.013	0.339	0.302	0.315	0.107	−0.012	0.156
			p	0.886	0.003	0.002	0.0004	0.269	0.898	0.090

Adjusted proportion of the variance explained by the model (Adj. R^2), standardized estimate (β), and levels of significance (p) from multiple linear regressions for dependent variables with significant correlations in Table S2 and potential confounders. Only data for ANOVA $p < 0.05$ are shown.

Table S4. Correlation analysis of age with lymphocyte markers.

	t1		t2		t3	
	r	p	r	p	r	p
Leukocytes (μL⁻¹)	0.075	0.231	0.026	0.698	−0.030	0.691
Lymphocytes (μL⁻¹)	0.012	0.851	−0.013	0.847	−0.114	0.131
Lymphocytes (% of leukocytes)	−0.100	0.110	−0.044	0.522	−0.128	0.089
CD19+ (μL⁻¹)	−0.202	0.001	−0.132	0.051	−0.218	0.004
CD19+ (% of lymphocytes)	−0.255	<0.001	−0.159	0.019	−0.205	0.006
CD19+CD5+ (% of CD19+)	−0.006	0.924	−0.190	0.005	−0.126	0.095
CD3+ (μL⁻¹)	0.004	0.954	0.005	0.941	−0.090	0.234
CD3+ (% of lymphocytes)	0.018	0.771	0.063	0.358	0.042	0.580
CD3+CD4+ (μL⁻¹)	0.042	0.501	0.031	0.648	−0.038	0.619
CD3+CD8+ (μL⁻¹)	−0.018	0.779	−0.044	0.520	−0.128	0.090
CD3+CD4+/CD3+CD8+	0.048	0.440	0.068	0.318	0.098	0.196
CD3+CD8+CD11b-/CD3+CD8+CD11b+	−0.171	0.006	−0.110	0.104	−0.046	0.542
CD3+CD8+CD11b- (μL⁻¹)	−0.024	0.704	−0.049	0.472	−0.068	0.367
CD3+CD8+CD11b+ (μL⁻¹)	0.162	0.009	0.097	0.155	−0.005	0.950
CD16+CD56+ (μL⁻¹)	0.126	0.044	0.098	0.148	0.031	0.680
CD3+HLA_DR+ (% of CD3+)	0.356	<0.001	0.450	<0.001	0.385	<0.001
CD3+CD25+ (% of CD3+)	0.325	<0.001	0.353	<0.001	0.367	<0.001

Pearson-correlation between immune cell phenotyping and age. Statistically significant ($p < 0.05$) positive correlations are highlighted in blue, negative ones in green.

Table S5. Correlation between age and PCB burden.

No. of Cl		PCB sum	PCB28	PCB101	PCB105	PCB114	PCB118	PCB138	PCB153	PCB156	PCB157	PCB167	PCB180	PCB189
			3	5				6					7	
t1	r	0.041	−0.256	−0.347	−0.227	−0.150	−0.172	0.010	0.103	0.168	0.075	−0.021	0.264	0.205
	p	0.516	<0.001	<0.001	<0.001	0.016	0.005	0.876	0.098	0.007	0.228	0.742	<0.001	<0.001
t2	r	0.082	−0.233	−0.350	−0.206	−0.096	−0.150	0.037	0.126	0.187	0.110	0.056	0.290	0.252
	p	0.232	<0.001	<0.001	0.002	0.159	0.027	0.587	0.065	0.006	0.108	0.410	<0.001	<0.001
t3	r	0.102	−0.174	−0.293	−0.188	−0.107	−0.141	0.031	0.123	0.180	0.112	0.052	0.287	0.238
	p	0.177	0.020	<0.001	0.012	0.156	0.061	0.680	0.102	0.016	0.139	0.488	<0.001	0.001

Pearson-correlation between PCB plasma levels and age. Statistically significant ($p < 0.05$) positive correlations are highlighted in blue, negative ones in green.

Table S6. Pearson correlation analysis of lymphocyte markers with PCB congener plasma levels in t1.

		PCB28	PCB101	PCB105	PCB114	PCB118	PCB138	PCB153	PCB156	PCB157	PCB167	PCB180	PCB189
No. of Cl		3	5				6					7	
Exposure (ng/g TSL)	Median	9.61	2.48	4.82	1.27	22.64	91.66	118.40	14.28	2.47	4.51	94.35	1.89
	25%	3.19	0.73	1.89	0.69	10.13	44.51	61.11	6.59	1.11	1.89	44.48	0.84
	75%	81.77	16.88	31.95	4.77	117.80	234.50	267.30	31.27	5.41	13.19	180.10	4.16
Leukocytes (μL⁻¹)	r	0.001	0.035	0.031	0.064	0.037	0.092	0.099	0.119	0.098	0.026	0.119	0.116
	p	0.984	0.581	0.622	0.302	0.552	0.143	0.111	0.057	0.116	0.674	0.056	0.064
Lymphocytes (μL⁻¹)	r	0.000	0.056	0.052	0.076	0.057	0.116	0.118	0.125	0.116	0.070	0.125	0.123
	p	0.999	0.371	0.409	0.226	0.362	0.063	0.059	0.046	0.062	0.262	0.046	0.048
Lymphocytes (% of leukocytes)	r	−0.031	0.023	−0.005	−0.019	−0.007	−0.018	−0.031	−0.039	−0.027	0.003	−0.048	−0.045
	p	0.619	0.708	0.934	0.767	0.914	0.770	0.622	0.529	0.668	0.960	0.445	0.476
CD19+ (μL⁻¹)	r	0.163	0.228	0.202	0.150	0.191	0.175	0.146	0.148	0.155	0.139	0.116	0.106
	p	0.009	<0.001	0.001	0.016	0.002	0.005	0.019	0.017	0.013	0.026	0.062	0.089
CD19+ (% of lymphocytes)	r	0.230	0.265	0.256	0.170	0.234	0.177	0.140	0.134	0.140	0.170	0.097	0.074
	p	<0.001	<0.001	<0.001	0.006	<0.001	0.004	0.025	0.032	0.024	0.006	0.120	0.234
CD19+CD5+ (% of CD19+)	r	−0.014	0.000	0.008	−0.018	0.006	−0.025	−0.032	−0.045	−0.076	−0.032	−0.052	−0.083
	p	0.823	0.995	0.893	0.771	0.928	0.694	0.605	0.468	0.224	0.608	0.402	0.182
CD3+ (μL⁻¹)	r	0.018	0.075	0.060	0.100	0.071	0.114	0.111	0.116	0.110	0.068	0.113	0.111
	p	0.774	0.227	0.339	0.109	0.253	0.067	0.076	0.062	0.077	0.279	0.069	0.074
CD3+ (% of lymphocytes)	r	0.000	−0.026	−0.042	0.012	−0.027	−0.030	−0.028	−0.034	−0.030	−0.030	−0.025	−0.032
	p	0.997	0.680	0.503	0.853	0.668	0.634	0.654	0.584	0.627	0.627	0.691	0.607
CD3+CD4+ (μL⁻¹)	r	0.019	0.063	0.051	0.101	0.064	0.112	0.116	0.131	0.115	0.061	0.127	0.127
	p	0.766	0.310	0.414	0.105	0.303	0.072	0.063	0.035	0.065	0.329	0.042	0.041
CD3+CD8+ (μL⁻¹)	r	−0.021	0.043	0.024	0.040	0.032	0.051	0.039	0.031	0.051	0.034	0.029	0.026
	p	0.734	0.488	0.701	0.526	0.610	0.417	0.533	0.622	0.411	0.586	0.639	0.683

Table S6. *Cont.*

		PCB28	PCB101	PCB105	PCB114	PCB118	PCB138	PCB153	PCB156	PCB157	PCB167	PCB180	PCB189
**No. of Cl**		**3**		**5**					**6**				**7**
CD3+CD4+/CD3+CD8+	r	0.036	0.007	0.017	0.042	0.020	0.038	0.053	0.073	0.040	0.014	0.070	0.075
	p	0.569	0.907	0.786	0.502	0.753	0.540	0.398	0.244	0.526	0.825	0.261	0.232
CD3+CD8+CD11b- /CD3+CD8+CD11b+	r	0.190	0.181	0.165	0.158	0.167	0.149	0.135	0.116	0.129	0.148	0.103	0.099
	p	0.002	0.004	0.008	0.011	0.007	0.017	0.031	0.065	0.040	0.018	0.101	0.114
CD3+CD8+CD11b- (μL⁻¹)	r	0.077	0.119	0.112	0.122	0.112	0.126	0.118	0.112	0.129	0.101	0.103	0.114
	p	0.217	0.057	0.073	0.050	0.072	0.043	0.059	0.072	0.039	0.105	0.099	0.068
CD3+CD8+CD11b+ (μL⁻¹)	r	−0.219	−0.163	−0.160	−0.130	−0.154	−0.103	−0.091	−0.077	−0.086	−0.127	−0.059	−0.058
	p	<0.001	0.009	0.010	0.036	0.013	0.100	0.145	0.220	0.167	0.041	0.346	0.357
CD16+CD56+ (μL⁻¹)	r	−0.135	−0.090	−0.085	−0.064	−0.078	−0.013	0.002	0.016	0.017	−0.031	0.024	0.045
	p	0.031	0.151	0.171	0.306	0.214	0.833	0.976	0.799	0.789	0.615	0.695	0.474
CD3+HLA_DR+ (μL⁻¹)	r	−0.153	−0.174	−0.108	−0.086	−0.097	−0.015	0.026	0.034	0.007	−0.015	0.084	0.047
	p	0.014	0.005	0.084	0.169	0.121	0.815	0.677	0.590	0.905	0.815	0.176	0.452
CD3+CD25+ (μL⁻¹)	r	−0.042	−0.129	−0.078	−0.045	−0.069	0.010	0.048	0.095	0.029	−0.038	0.123	0.105
	p	0.501	0.038	0.214	0.471	0.269	0.876	0.441	0.126	0.647	0.538	0.048	0.093

Pearson-correlation between plasma PCB congener levels and immune cell phenotyping. Statistically significant ($p < 0.05$) positive correlations are highlighted in blue, negative ones in green.

Table S7. Multiple linear regression for significant correlations from Table S6.

Dependent Variable	Congener	Adj. R^2	Independent Variables													
			PCB		Age		Smoking		Pack Years		Sex		BMI		Alcohol	
			β	p	β	p	β	p	β	p	β	p	β	p	β	p
CD19 (μL^{-1})	28	0.156	0.102	0.153	−0.222	0.016	0.211	0.007	0.140	0.093	−0.066	0.346	0.137	0.054	−0.120	0.092
	101	0.170	0.166	0.026	−0.187	0.046	0.210	0.007	0.135	0.103	−0.053	0.447	0.142	0.045	−0.118	0.096
	105	0.165	0.143	0.049	−0.211	0.021	0.207	0.008	0.141	0.088	−0.052	0.464	0.145	0.041	−0.128	0.072
	114	0.151	0.065	0.363	−0.236	0.010	0.211	0.008	0.140	0.093	−0.070	0.324	0.145	0.044	−0.125	0.085
	118	0.164	0.135	0.058	−0.219	0.016	0.208	0.008	0.142	0.086	−0.059	0.404	0.147	0.039	−0.130	0.069
	138	0.162	0.126	0.075	−0.242	0.007	0.205	0.009	0.138	0.095	−0.055	0.434	0.153	0.033	−0.130	0.069
	153	0.160	0.119	0.092	−0.253	0.005	0.206	0.009	0.138	0.097	−0.058	0.416	0.154	0.032	−0.130	0.070
	156	0.164	0.137	0.057	−0.264	0.003	0.201	0.011	0.134	0.106	−0.056	0.431	0.160	0.026	−0.131	0.068
	157	0.163	0.132	0.064	−0.255	0.005	0.206	0.009	0.137	0.098	−0.055	0.437	0.162	0.025	−0.125	0.078
	167	0.158	0.107	0.127	−0.240	0.008	0.209	0.008	0.146	0.079	−0.064	0.369	0.148	0.038	−0.132	0.066
CD19 (%)	28	0.165	0.172	0.016	−0.220	0.016	0.140	0.072	0.095	0.252	−0.080	0.256	0.137	0.053	−0.161	0.024
	101	0.173	0.202	0.007	−0.191	0.041	0.140	0.070	0.091	0.270	−0.070	0.322	0.143	0.043	−0.155	0.028
	105	0.178	0.210	0.004	−0.210	0.021	0.135	0.081	0.098	0.232	−0.061	0.387	0.150	0.034	−0.172	0.016
	114	0.152	0.126	0.081	−0.241	0.009	0.139	0.077	0.094	0.256	−0.083	0.239	0.152	0.035	−0.170	0.019
	118	0.174	0.196	0.006	−0.223	0.014	0.137	0.078	0.099	0.226	−0.071	0.308	0.152	0.032	−0.173	0.015
	138	0.162	0.161	0.023	−0.258	0.004	0.134	0.087	0.095	0.252	−0.071	0.319	0.158	0.028	−0.171	0.017
	153	0.159	0.150	0.034	−0.271	0.003	0.136	0.083	0.094	0.255	−0.074	0.298	0.159	0.027	−0.171	0.018
	156	0.163	0.164	0.023	−0.284	0.002	0.130	0.098	0.090	0.275	−0.073	0.304	0.165	0.022	−0.171	0.017
	157	0.158	0.148	0.038	−0.273	0.003	0.137	0.081	0.094	0.255	−0.074	0.299	0.166	0.022	−0.164	0.022
	167	0.165	0.168	0.017	−0.251	0.005	0.137	0.079	0.105	0.202	−0.077	0.276	0.155	0.30	−0.179	0.013
CD3+CD4+ (μL^{-1})	156	0.062	0.057	0.455	−0.009	0.927	0.243	0.004	0.144	0.102	0.079	0.290	0.066	0.386	0.070	0.351
	180	0.062	0.058	0.453	−0.014	0.879	0.244	0.004	0.143	0.103	0.081	0.284	0.068	0.374	0.071	0.345
	189	0.061	0.049	0.532	−0.012	0.901	0.243	0.004	0.143	0.104	0.079	0.294	0.067	0.383	0.072	0.336
CD3+CD8+CD11b+ (μL^{-1})	28	0.039	−0.250	0.001	0.080	0.412	0.040	0.633	−0.100	0.259	−0.049	0.511	0.025	0.741	0.032	0.673
CD16+CD56+ (μL^{-1})	28	0.054	−0.151	0.046	0.137	0.157	−0.003	0.970	−0.083	0.343	−0.150	0.046	−0.044	0.560	0.152	0.044
CD3+HLA_DR+ (μL^{-1})	28	0.107	−0.136	0.065	0.322	0.001	−0.046	0.564	−0.087	0.306	−0.099	0.175	0.011	0.880	−0.046	0.531
	101	0.102	−0.123	0.109	0.313	0.001	−0.048	0.552	−0.087	0.312	−0.101	0.168	0.007	0.922	−0.051	0.490
CD3+CD25+ (μL^{-1})	101	0.205	−0.042	0.557	0.309	0.001	0.281	0.000	0.196	0.016	0.107	0.122	0.066	0.337	0.029	0.671
	180	0.204	−0.013	0.855	0.327	0.000	0.281	0.000	0.195	0.017	0.111	0.110	0.064	0.359	0.030	0.666

Adjusted proportion of the variance explained by the model (Adj. R^2), standardized estimate (β), and levels of significance (p) from multiple linear regressions for dependent variables with significant correlations in Table S6 and potential confounders. Only data for ANOVA $p < 0.05$ are shown.

Table S8. Pearson correlation analysis of lymphocyte markers with PCB congener plasma levels in t2.

		PCB28	PCB101	PCB105	PCB114	PCB118	PCB138	PCB153	PCB156	PCB157	PCB167	PCB180	PCB189
**No. of Cl**		3	5				6					7	
Exposure (ng/g TSL)	Median	10.14	2.96	7.42	1.74	32.25	99.93	131.50	14.77	2.58	4.84	97.02	2.08
	25%	3.11	0.80	2.38	0.72	11.29	51.87	73.59	7.33	1.13	2.04	48.75	0.85
	75%	63.89	10.45	35.45	7.36	141.60	353.30	387.80	43.32	7.87	17.18	238.30	5.01
Leukocytes (μL⁻¹)	r	0.004	0.014	0.048	0.079	0.053	0.096	0.087	0.097	0.095	0.072	0.077	0.097
	p	0.952	0.843	0.485	0.246	0.440	0.161	0.201	0.155	0.166	0.294	0.260	0.154
Lymphocytes (μL⁻¹)	r	0.090	0.088	0.112	0.128	0.114	0.115	0.092	0.092	0.098	0.097	0.054	0.059
	p	0.187	0.196	0.100	0.061	0.094	0.093	0.180	0.179	0.149	0.154	0.432	0.386
Lymphocytes (% of leukocytes)	r	0.101	0.086	0.076	0.057	0.073	0.024	0.007	−0.003	0.007	0.032	−0.023	−0.040
	p	0.141	0.211	0.268	0.406	0.288	0.727	0.915	0.967	0.918	0.644	0.736	0.558
CD19+ (μL⁻¹)	r	0.096	0.121	0.161	0.131	0.147	0.110	0.074	0.083	0.086	0.087	0.027	0.035
	p	0.159	0.076	0.018	0.054	0.031	0.106	0.278	0.226	0.208	0.204	0.688	0.606
CD19+ (% of lymphocytes)	r	0.079	0.113	0.137	0.079	0.114	0.069	0.038	0.047	0.042	0.050	0.008	0.008
	p	0.249	0.099	0.044	0.247	0.094	0.313	0.577	0.495	0.538	0.469	0.909	0.901
CD19+CD5+ (% of CD19+)	r	0.110	0.012	−0.007	−0.073	−0.030	−0.136	−0.163	−0.192	−0.165	−0.117	−0.232	−0.229
	p	0.106	0.857	0.924	0.286	0.656	0.045	0.016	0.005	0.015	0.087	<0.001	<0.001
CD3+ (μL⁻¹)	r	0.106	0.086	0.105	0.118	0.106	0.099	0.079	0.080	0.085	0.084	0.044	0.046
	p	0.119	0.208	0.122	0.085	0.121	0.148	0.247	0.243	0.213	0.218	0.519	0.500
CD3+ (% of lymphocytes)	r	0.071	0.023	0.018	0.018	0.016	−0.001	0.002	0.002	−0.001	0.003	−0.004	−0.017
	p	0.297	0.742	0.796	0.794	0.818	0.987	0.980	0.975	0.991	0.969	0.958	0.805
CD3+CD4+ (μL⁻¹)	r	0.091	0.052	0.098	0.127	0.099	0.098	0.085	0.096	0.085	0.083	0.063	0.071
	p	0.180	0.448	0.150	0.062	0.147	0.149	0.212	0.159	0.213	0.223	0.357	0.297
CD3+CD8+ (μL⁻¹)	r	0.075	0.093	0.049	0.041	0.051	0.030	0.007	−0.004	0.024	0.026	−0.032	−0.035
	p	0.275	0.173	0.476	0.551	0.455	0.656	0.922	0.948	0.728	0.705	0.635	0.605
CD3+CD4+/CD3+CD8+	r	−0.006	−0.055	0.027	0.060	0.026	0.047	0.061	0.081	0.043	0.040	0.083	0.093
	p	0.929	0.422	0.688	0.379	0.703	0.493	0.374	0.237	0.526	0.562	0.225	0.174
CD3+CD8+CD11b-/CD3+CD8+CD11b+	r	0.078	0.150	0.081	0.055	0.071	0.037	0.017	0.004	0.007	0.025	−0.010	−0.035
	p	0.251	0.028	0.234	0.422	0.297	0.586	0.801	0.950	0.921	0.719	0.884	0.607
CD3+CD8+CD11b- (μL⁻¹)	r	0.074	0.138	0.079	0.073	0.080	0.073	0.051	0.033	0.049	0.057	0.017	0.006
	p	0.278	0.043	0.250	0.283	0.243	0.288	0.454	0.633	0.470	0.402	0.809	0.926
CD3+CD8+CD11b+(μL⁻¹)	r	−0.039	−0.067	−0.035	−0.004	−0.020	0.020	0.026	0.026	0.039	0.023	0.030	0.052
	p	0.567	0.325	0.611	0.958	0.767	0.771	0.705	0.709	0.569	0.740	0.659	0.443
CD16+CD56+ (μL⁻¹)	r	−0.043	−0.011	0.005	0.040	0.021	0.072	0.077	0.070	0.075	0.070	0.078	0.092
	p	0.534	0.874	0.942	0.561	0.757	0.293	0.262	0.306	0.271	0.305	0.254	0.176
CD3+HLA_DR+ (μL⁻¹)	r	−0.173	−0.147	−0.121	−0.071	−0.083	0.024	0.068	0.094	0.072	0.044	0.144	0.112
	p	0.011	0.031	0.075	0.296	0.223	0.722	0.322	0.169	0.289	0.516	0.035	0.099

Table S8. *Cont.*

		PCB28	PCB101	PCB105	PCB114	PCB118	PCB138	PCB153	PCB156	PCB157	PCB167	PCB180	PCB189
No. of Cl		3	5				6					7	
CD3+CD25+ (µL^{-1})	r	−0.018	−0.068	0.030	0.114	0.049	0.151	0.183	0.220	0.201	0.130	0.244	0.255
	p	0.796	0.317	0.666	0.095	0.478	0.026	0.007	0.001	0.003	0.056	<0.001	<0.001

Pearson-correlation between plasma PCB congener levels and immune cell phenotyping. Statistically significant ($p < 0.05$) positive correlations are highlighted in blue, negative ones in green.

Table S9. Multiple linear regression for significant correlations from Table S8.

Dependent Variable	Congener	Adj. R²	Independent Variables													
			PCB		Age		Smoking		Pack Years		Sex		BMI		Alcohol	
			β	p	β	p	β	p	β	p	β	p	β	p	β	p
CD19 (µL^{-1})	105	0.167	0.125	0.122	−0.076	0.428	0.318	0.000	0.131	0.154	−0.039	0.636	0.152	0.066	−0.197	0.014
	118	0.167	0.121	0.129	−0.082	0.394	0.320	0.000	0.130	0.161	−0.043	0.605	0.154	0.063	−0.198	0.014
CD19 (%)	105	0.148	0.086	0.293	−0.161	0.100	0.188	0.031	0.152	0.105	−0.112	0.183	0.152	0.069	−0.242	0.003
CD19+CD5+ (% of CD19+)	138	0.093	−0.113	0.178	−0.309	0.002	−0.072	0.422	0.033	0.732	0.137	0.115	0.214	0.013	0.020	0.807
	153	0.095	−0.122	0.146	−0.299	0.003	−0.072	0.421	0.034	0.726	0.136	0.115	0.212	0.014	0.023	0.783
	156	0.098	−0.136	0.110	−0.288	0.004	−0.068	0.449	0.037	0.696	0.134	0.120	0.208	0.016	0.022	0.791
	157	0.095	−0.123	0.148	−0.295	0.003	−0.066	0.458	0.033	0.733	0.133	0.125	0.208	0.017	0.020	0.807
	180	0.103	−0.160	0.069	−0.267	0.009	−0.069	0.434	0.039	0.687	0.129	0.134	0.201	0.020	0.028	0.741
	189	0.102	−0.156	0.079	−0.271	0.008	−0.062	0.486	0.045	0.642	0.134	0.118	0.199	0.022	0.029	0.732
CD3+HLA_DR+ (µL^{-1})	28	0.146	−0.104	0.203	0.416	0.000	0.024	0.783	−0.252	0.008	−0.141	0.090	−0.039	0.642	0.106	0.193
	101	0.136	0.018	0.829	0.450	0.000	0.021	0.811	−0.257	0.007	−0.133	0.114	−0.059	0.484	0.096	0.236
CD3+CD25+(µL^{-1})	138	0.292	0.136	0.066	0.363	0.000	0.237	0.003	0.284	0.001	0.126	0.100	−0.074	0.332	−0.012	0.869
	153	0.291	0.133	0.074	0.351	0.000	0.237	0.003	0.284	0.001	0.124	0.106	−0.072	0.346	−0.014	0.851
	156	0.294	0.147	0.052	0.340	0.000	0.233	0.004	0.280	0.001	0.126	0.100	−0.067	0.378	−0.013	0.862
	157	0.297	0.157	0.037	0.345	0.000	0.229	0.004	0.283	0.001	0.132	0.086	−0.065	0.392	−0.013	0.861
	180	0.290	0.135	0.084	0.327	0.000	0.237	0.003	0.282	0.001	0.125	0.104	−0.063	0.408	−0.015	0.842
	189	0.295	0.155	0.049	0.325	0.000	0.229	0.004	0.273	0.002	0.124	0.104	−0.059	0.437	−0.018	0.805

Adjusted proportion of the variance explained by the model (Adj. R^2), standardized estimate (β), and levels of significance (p) from multiple linear regressions for dependent variables with significant correlations in Table S8 and potential confounders. Only data for ANOVA $p < 0.05$ are shown.

Table S10. Pearson correlation analysis of lymphocyte markers with PCB congener plasma levels in t3.

		PCB28	PCB101	PCB105	PCB114	PCB118	PCB138	PCB153	PCB156	PCB157	PCB167	PCB180	PCB189
**No. of Cl**		3	5				6					7	
Exposure (ng/g TSL)	Median	9.59	2.56	7.80	0.95	33.23	113.80	150.50	14.89	2.86	4.88	110.80	2.11
	25%	2.78	0.72	2.63	0.69	11.84	64.41	87.87	7.95	1.49	2.66	65.61	0.90
	75%	48.22	9.20	32.24	6.48	116.00	403.20	395.20	36.64	7.50	15.04	264.00	4.98
Leukocytes (µL⁻¹)	r	−0.113	−0.017	0.017	0.066	0.034	0.112	0.109	0.103	0.110	0.063	0.094	0.126
	p	0.135	0.824	0.818	0.385	0.654	0.138	0.150	0.172	0.144	0.403	0.215	0.094
Lymphocytes (µL⁻¹)	r	0.026	0.127	0.120	0.144	0.135	0.176	0.163	0.143	0.143	0.133	0.120	0.131
	p	0.730	0.091	0.111	0.055	0.073	0.019	0.030	0.058	0.058	0.078	0.112	0.082
Lymphocytes (% of leukocytes)	r	−0.050	−0.023	−0.082	−0.037	−0.084	−0.093	−0.089	−0.078	−0.095	−0.083	−0.082	−0.090
	p	0.507	0.757	0.278	0.621	0.269	0.216	0.238	0.300	0.207	0.273	0.279	0.236
CD19+ (µL⁻¹)	r	0.033	0.168	0.129	0.122	0.142	0.130	0.099	0.079	0.078	0.086	0.046	0.043
	p	0.662	0.025	0.086	0.107	0.060	0.084	0.188	0.296	0.304	0.257	0.545	0.566
CD19+ (% of lymphocytes)	r	0.043	0.137	0.093	0.052	0.097	0.044	0.013	0.000	−0.001	0.019	−0.027	−0.045
	p	0.567	0.069	0.218	0.489	0.198	0.562	0.859	0.995	0.987	0.798	0.716	0.551
CD19+CD5+ (% of CD19+)	r	0.092	0.018	−0.017	−0.025	−0.017	−0.072	−0.083	−0.098	−0.087	−0.050	−0.110	−0.125
	p	0.226	0.809	0.825	0.737	0.818	0.341	0.274	0.194	0.252	0.509	0.146	0.097
CD3+ (µL⁻¹)	r	0.046	0.128	0.134	0.173	0.151	0.187	0.173	0.161	0.151	0.152	0.134	0.138
	p	0.540	0.089	0.076	0.021	0.045	0.013	0.021	0.033	0.045	0.043	0.076	0.067
CD3+ (% of lymphocytes)	r	0.059	0.033	0.065	0.114	0.074	0.077	0.076	0.092	0.065	0.087	0.075	0.060
	p	0.435	0.664	0.393	0.131	0.328	0.311	0.316	0.222	0.391	0.250	0.321	0.427
CD3+CD4+ (µL⁻¹)	r	0.020	0.060	0.100	0.178	0.116	0.164	0.160	0.165	0.143	0.133	0.144	0.173
	p	0.792	0.425	0.187	0.017	0.125	0.030	0.034	0.028	0.057	0.077	0.057	0.021
CD3+CD8+ (µL⁻¹)	r	0.082	0.192	0.131	0.110	0.140	0.137	0.114	0.090	0.098	0.118	0.059	0.044
	p	0.277	0.011	0.082	0.146	0.063	0.069	0.130	0.232	0.193	0.116	0.433	0.565
CD3+CD4+/CD3+CD8+	r	−0.068	−0.148	−0.061	0.018	−0.058	−0.021	−0.001	0.026	0.003	−0.023	0.041	0.077
	p	0.367	0.050	0.424	0.816	0.446	0.785	0.992	0.732	0.967	0.757	0.586	0.309
CD3+CD8+CD11b-/CD3+CD8+CD11b+	r	0.063	0.108	0.080	0.076	0.071	0.046	0.034	0.024	0.036	0.057	0.025	0.014
	p	0.408	0.152	0.292	0.313	0.346	0.539	0.651	0.752	0.636	0.449	0.742	0.858
CD3+CD8+CD11b-(µL⁻¹)	r	0.086	0.207	0.150	0.142	0.157	0.163	0.147	0.124	0.129	0.147	0.113	0.083
	p	0.257	0.006	0.046	0.059	0.037	0.031	0.051	0.101	0.087	0.051	0.135	0.275
CD3+CD8+CD11b+(µL⁻¹)	r	−0.002	0.058	0.035	0.038	0.055	0.098	0.101	0.092	0.080	0.067	0.081	0.068
	p	0.981	0.444	0.642	0.619	0.471	0.193	0.183	0.224	0.293	0.377	0.282	0.369
CD16+CD56+(µL⁻¹)	r	−0.069	−0.026	−0.063	−0.057	−0.061	−0.012	−0.004	−0.018	0.003	−0.034	−0.006	0.030
	p	0.362	0.728	0.402	0.451	0.422	0.876	0.955	0.808	0.972	0.650	0.940	0.692
CD3+HLA_DR+(µL⁻¹)	r	−0.149	−0.133	−0.110	−0.114	−0.094	−0.008	0.028	0.035	0.004	0.005	0.085	0.017
	p	0.048	0.078	0.146	0.131	0.215	0.915	0.713	0.640	0.956	0.943	0.260	0.820

Table S10. *Cont.*

		PCB28	PCB101	PCB105	PCB114	PCB118	PCB138	PCB153	PCB156	PCB157	PCB167	PCB180	PCB189
**No. of Cl**		**3**		**5**					**6**			**7**	
CD3+CD25+(μL^{-1})	r	−0.034	−0.092	0.014	0.121	0.024	0.136	0.174	0.211	0.192	0.115	0.245	0.265
	p	0.653	0.224	0.854	0.107	0.749	0.071	0.020	0.005	0.011	0.128	0.001	<0.001

Pearson-correlation between plasma PCB congener levels and immune cell phenotyping. Statistically significant ($p < 0.05$) positive correlations are highlighted in blue, negative ones in green.

Table S11. Multiple linear regression for significant correlations from Table S10.

Dependent Variable	Congener	Adj. R^2	Independent Variables													
			PCB		Age		Smoking		Pack years		Sex		BMI		Alcohol	
			β	p	β	p	β	p	β	p	β	p	β	p	β	p
CD19 (%)	101	0.133	0.061	0.569	−0.273	0.041	0.148	0.178	0.005	0.967	−0.126	0.246	0.218	0.048	−0.136	0.188
CD3+CD4+ (μL^{-1})	114	0.082	0.112	0.316	−0.101	0.450	0.252	0.027	0.168	0.18	0.087	0.436	0.046	0.682	0.161	0.129
	138	0.081	0.103	0.338	−0.115	0.380	0.256	0.025	0.178	0.158	0.085	0.447	0.049	0.664	0.157	0.141
	153	0.081	0.103	0.337	−0.123	0.343	0.257	0.024	0.177	0.160	0.084	0.450	0.051	0.649	0.156	0.144
	156	0.085	0.124	0.256	−0.131	0.309	0.253	0.026	0.170	0.179	0.088	0.429	0.053	0.635	0.152	0.153
	189	0.093	0.160	0.153	−0.148	0.253	0.259	0.022	0.157	0.214	0.092	0.408	0.065	0.558	0.151	0.155
CD3+HLA_DR+ (μL^{-1})	28	0.152	−0.197	0.058	0.385	0.003	0.055	0.609	−0.300	0.013	−0.185	0.083	0.092	0.406	0.010	0.921
CD3+CD25+ (μL^{-1})	153	0.316	0.017	0.857	0.339	0.003	0.303	0.002	0.314	0.005	0.107	0.266	−0.012	0.902	0.155	0.092
	156	0.316	0.015	0.870	0.338	0.003	0.302	0.002	0.314	0.005	0.107	0.267	−0.012	0.904	0.155	0.093
	157	0.315	0.002	0.982	0.338	0.003	0.303	0.002	0.317	0.004	0.105	0.280	−0.012	0.903	0.157	0.087
	180	0.316	0.013	0.892	0.337	0.003	0.303	0.002	0.314	0.005	0.107	0.269	−0.011	0.909	0.156	0.091
	189	0.316	−0.015	0.875	0.339	0.003	0.304	0.002	0.321	0.004	0.102	0.288	−0.013	0.891	0.159	0.084

Adjusted proportion of the variance explained by the model (Adj. R^2), standardized estimate (β), and levels of significance (p) from multiple linear regressions for dependent variables with significant correlations in Table S10 and potential confounders. Only data for ANOVA $p < 0.05$ are shown.

Table S12. Correlation analysis of PCB congeners with antibody levels.

	No. of Cl		Total PCB	PCB28	PCB101	PCB105	PCB114	PCB118	PCB138	PCB153	PCB156	PCB157	PCB167	PCB180	PCB189
				3			5				6			7	
t1	IgG	r	0.011	0.034	0.004	0.016	0.005	0.035	0.013	0.007	−0.002	−0.008	0.022	0.013	0.000
		p	0.858	0.587	0.952	0.794	0.942	0.571	0.837	0.911	0.976	0.901	0.730	0.839	0.996
	IgA	r	0.037	0.057	−0.019	0.007	0.014	0.013	0.023	0.035	0.038	0.033	0.023	0.063	0.032
		p	0.551	0.363	0.767	0.907	0.821	0.831	0.711	0.575	0.539	0.594	0.714	0.310	0.609
	IgM	r	−0.146	−0.158	−0.131	−0.161	−0.189	−0.145	−0.146	−0.138	−0.132	−0.158	−0.169	−0.127	−0.136
		p	0.019	0.011	0.035	0.010	0.002	0.020	0.019	0.026	0.034	0.011	0.007	0.041	0.028
t2	IgG	r	0.004	−0.017	−0.015	−0.008	0.033	0.014	−0.001	−0.006	−0.011	0.004	0.026	−0.002	−0.002
		p	0.953	0.809	0.831	0.902	0.629	0.842	0.983	0.932	0.873	0.958	0.708	0.977	0.977
	IgA	r	0.095	0.026	−0.017	0.052	0.080	0.071	0.088	0.097	0.100	0.109	0.111	0.132	0.117
		p	0.164	0.706	0.807	0.445	0.246	0.304	0.198	0.157	0.146	0.111	0.105	0.054	0.087
	IgM	r	−0.147	−0.121	−0.108	−0.138	−0.149	−0.143	−0.159	−0.160	−0.152	−0.142	−0.160	−0.152	−0.158
		p	0.032	0.077	0.116	0.044	0.029	0.037	0.020	0.020	0.026	0.038	0.019	0.026	0.020
t3	IgG	r	−0.050	−0.018	−0.099	−0.076	−0.023	−0.060	−0.061	−0.057	−0.070	−0.031	−0.010	−0.052	−0.020
		p	0.508	0.808	0.191	0.313	0.761	0.430	0.417	0.448	0.354	0.679	0.899	0.491	0.791
	IgA	r	0.078	0.057	−0.020	0.073	0.109	0.070	0.075	0.075	0.075	0.111	0.092	0.092	0.138
		p	0.300	0.451	0.794	0.333	0.148	0.355	0.319	0.322	0.320	0.142	0.222	0.224	0.066
	IgM	r	−0.058	−0.052	−0.067	−0.077	−0.075	−0.069	−0.071	−0.072	−0.080	−0.036	−0.048	−0.058	−0.104
		p	0.444	0.489	0.379	0.308	0.320	0.360	0.351	0.341	0.293	0.633	0.526	0.440	0.170

Pearson-correlation between plasma PCB levels and serum antibody levels. Statistically significant ($p < 0.05$) positive correlations are highlighted in blue, negative ones in green.

Table S13. Multiple linear regression for significant correlations from Table S12.

Dependent Variable	t	Congener	Adj. R^2	Independent Variables													
				PCB		Age		Smoking		Pack Years		Sex		BMI		Alcohol	
				β	p	β	p	β	p	β	p	β	p	β	p	β	p
IgM	1	114	0.039	−0.205	0.008	−0.186	0.055	−0.141	0.093	0.183	0.040	0.052	0.493	−0.038	0.613	0.091	0.233

Adjusted proportion of the variance explained by the model (Adj. R^2), standardized estimate (β), and levels of significance (p) from multiple linear regressions for dependent variables with significant correlations in Table S12 and potential confounders. Only data for ANOVA $p < 0.05$ are shown.

Table S14. Correlation analysis of PCB congeners with IFN-γ production in t1.

No. of Cl		Total PCB	PCB28	PCB101	PCB105	PCB114	PCB118	PCB138	PCB153	PCB156	PCB157	PCB167	PCB180	PCB189
			3	5				6					7	
72h PHA	r	−0.007	−0.078	−0.038	−0.020	−0.021	−0.011	0.008	0.016	0.013	0.011	0.013	0.034	0.028
	p	0.922	0.243	0.564	0.765	0.755	0.865	0.907	0.805	0.844	0.874	0.846	0.614	0.676
72h SPEA	r	−0.012	0.004	0.051	0.000	0.036	−0.003	−0.004	−0.015	−0.013	0.002	−0.026	−0.029	0.029
	p	0.854	0.957	0.447	0.995	0.594	0.963	0.947	0.826	0.847	0.980	0.700	0.661	0.664
120h SPEA	r	−0.017	0.014	0.074	0.005	0.044	0.007	−0.009	−0.029	−0.026	−0.012	−0.019	−0.049	−0.007
	p	0.796	0.834	0.268	0.945	0.514	0.919	0.890	0.667	0.696	0.863	0.780	0.461	0.912

Pearson-correlation between plasma PCB levels and IFN-γ production.

Table S15. Correlation analysis of PCB congeners with markers of T- and NK-cell activation in t3.

No. of Cl		Total PCB	PCB28	PCB101	PCB105	PCB114	PCB118	PCB138	PCB153	PCB156	PCB157	PCB167	PCB180	PCB189
			3	5				6					7	
CD3+CD45RA+ (μL^{-1})	r	−0.080	0.004	−0.011	−0.022	−0.085	−0.005	−0.046	−0.052	−0.074	−0.084	−0.024	−0.086	−0.076
	p	0.295	0.959	0.887	0.772	0.264	0.952	0.542	0.495	0.332	0.270	0.748	0.257	0.315
CD3+CD45R0+ (μL^{-1})	r	0.152	0.056	0.096	0.084	0.105	0.094	0.136	0.144	0.174	0.192	0.153	0.158	0.155
	p	0.045	0.458	0.206	0.271	0.167	0.218	0.073	0.058	0.022	0.011	0.044	0.036	0.040
CD3+CD16+/56+ (% of lymphocytes)	r	0.123	0.068	0.063	0.081	0.013	0.103	0.103	0.116	0.098	0.090	0.111	0.113	0.054
	p	0.104	0.371	0.409	0.288	0.861	0.176	0.173	0.128	0.195	0.238	0.145	0.136	0.479
CD3+CD16+/56+ (μL^{-1})	r	0.165	0.075	0.104	0.118	0.064	0.144	0.158	0.165	0.143	0.135	0.151	0.149	0.096
	p	0.029	0.326	0.170	0.121	0.399	0.058	0.036	0.029	0.060	0.075	0.046	0.050	0.204
NK-cell activity	r	−0.075	−0.028	−0.086	−0.084	−0.083	−0.092	−0.083	−0.064	−0.054	−0.063	−0.090	−0.040	0.002
	p	0.329	0.712	0.259	0.272	0.276	0.230	0.276	0.400	0.482	0.408	0.240	0.601	0.982

Pearson-correlation between plasma PCB levels and markers of T- and NK-cell activation. Statistically significant ($p < 0.05$) positive correlations are highlighted in blue.

© 2016 by the authors; licensee MDPI, Basel, Switzerland. This article is an open access article distributed under the terms and conditions of the Creative Commons by Attribution (CC-BY) license (http://creativecommons.org/licenses/by/4.0/).
